# Supplementary figures and images for: MBNL1 alternative splicing isoforms play opposing roles in cancer
Source: Life Sci Alliance. 2018 Sep 7;1(5):e201800157. doi: 10.26508/lsa.201800157 (PMC6238595; doi:10.26508/lsa.201800157)

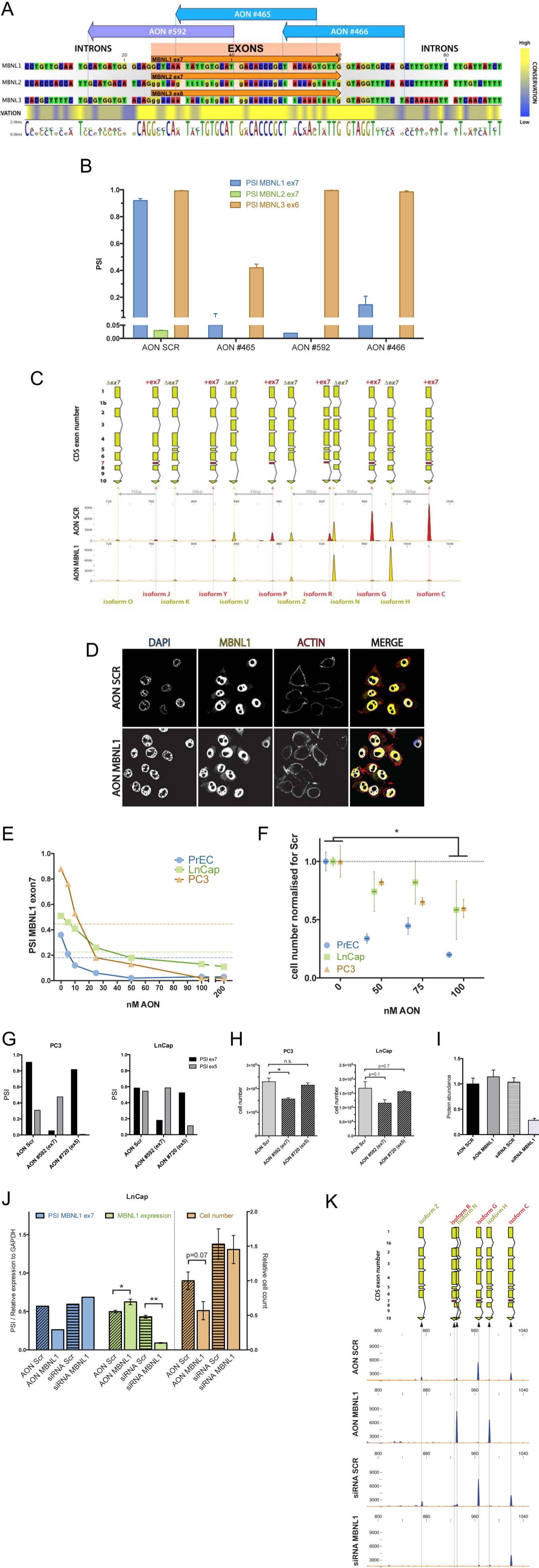

Supplement: Supplementary file 3 [file LSA-2018-00157_FigS3.pdf]
